# Challenging times in medical education in the era of artificial intelligence can provide opportunities

**DOI:** 10.1016/j.clinme.2025.100536

**Published:** 2025-11-20

**Authors:** Aftab Ala, Ponnusamy Saravanan

**Affiliations:** a Institute of Liver Studies, King’s College Hospital NHS Foundation Trust, London, UK; b Diabetes, Endocrinology and Metabolism, George Eliot Hospital NHS Trust, Nuneaton, UK; c Warwick Applied Health, Warwick Medical School, Centre for Global Health and Centre for Early Life, University of Warwick, Coventry, UK

Rapid advances in evidence-based care across all areas of medicine are good news for patients and for population health. Health systems worldwide have increasingly adopted clinical guidelines to help healthcare professionals deliver the best available care. Established bodies such as the National Institute for Health and Care Excellence (NICE) also provide patients with a clear benchmark for what constitutes an acceptable standard of care.

Yet keeping guidelines up to date in an era of rapid scientific progress presents significant challenges – both for experienced clinicians and for those still in training. This issue of *Clinical Medicine* explores several of these challenges within hepatology. I am delighted to co-author this issue’s editorial with Prof Ala. Together with Prof Saxena, two of the country’s leading hepatologists, they have carefully commissioned an excellent collection of articles on liver disorders. I am confident that these papers will complement existing guidelines and offer updated learning for both generalists and specialists. A summary of these articles follows.

Liver disease remains a major global health challenge in diagnosis and management and a major cause of death and disability. Morbidity and mortality are significant due to the high prevalence of undiagnosed disease, with many cases only identified at late stages when treatment options are limited, and complications such as cirrhosis and hepatocellular carcinoma can lead to life-threatening disease burden.^1^^,^^2^ The hepatology CME section of this issue of *Clinical Medicine* addresses the diverse presentations of liver disease with evidence-based guidance for practising clinicians to raise awareness, building knowledge to guide early diagnosis and management decision making for internal medicine clinicians who remain key gatekeepers to specialist hepatology services.

Mathew *et al*^3^ critically review and update on risk factors related to hepatocellular carcinoma, surveillance, key features on diagnosis, treatment and management decision making. Farooq *et al*^4^ describe key features of diagnosis of hepatitis C and major treatment updates in enabling cure, tackling health inequality and WHO call for future total global elimination. Watson *et al*^5^ outline the main key features of hepatitis B including dissecting the diagnosis pathways, new biomarkers, current and future exciting treatment options.

Hsu *et al*^6^ carefully describe metabolic dysfunction-associated steatosis liver disease (MASLD) pathogenesis, natural history, diagnosis and management including GLP-1 therapies. The article by Mansour^7^ discusses the importance of the early recognition, new terminologies and principles of management of acute decompensated cirrhosis and acute on chronic liver failure.

Bains *et al*^8^ outline the key paediatric liver diseases relevant to adult practice, highlighting fundamental of care and long-term considerations for this unique and often complex patient population. Paediatric chronic liver diseases are rare, but are increasingly encountered in adult practice as survival improves and more adolescents transition to adult services.

Aloumi *et al*^9^ provide a robust practical update on recognising, investigating and managing rare genetic liver conditions, aiming to support much earlier diagnosis, better patient outcomes, and improved use of genomic services in frontline practice.

Cussen *et al*^10^ summarise the challenging field of palliative care in people with advanced liver disorders. They highlight the importance of recognising the challenges, new developments in prognostication and the need for careful advance care planning for patients with chronic liver disease.

Finally, I would like to highlight the systematic review by Leong *et al*^11^ as my personal choice from the rest of the issue. This systematic review summarises the current evidence, outlines the difficulties that clinicians face in adhering to and implementing evolving guidelines and new evidence in everyday practice. There is still a long way to go. Nevertheless, I remain optimistic. In this rapidly evolving era of artificial intelligence, co-developed clinical decision-support systems offer a promising opportunity to help us deliver the best possible care for the patient sitting in front of us. Every healthcare professional has responsibility in developing such systems.

## Declaration of competing interest

AA is an associate editor and PS is the editor-in-chief of *Clinical Medicine*.
